# Biochemical characterization of extracellular polymeric substances from endodontic biofilms

**DOI:** 10.1371/journal.pone.0204081

**Published:** 2018-11-20

**Authors:** Tatiana Ramirez-Mora, Cristina Retana-Lobo, Grettel Valle-Bourrouet

**Affiliations:** 1 Section of Endodontics, Restorative Department, Faculty of Dentistry, University of Costa Rica, Montes de Oca, San José, Costa Rica; 2 Inorganic Chemistry Department, Chemistry Faculty, University of Costa Rica, Montes de Oca, San José, Costa Rica; Laurentian, CANADA

## Abstract

Apical periodontitis is frequently associated with the presence of bacteria biofilm, which has an indisputable impact on the prognosis of endodontic therapy due to the high resistance to adverse environmental conditions, chemicals, and antibiotic therapy that characterize bacteria within biofilm. The biofilm matrix acts as a protective shield over the encased microorganisms. The aim of this investigation was to identify the main biochemical components of biofilm matrix from endodontic mono- and dual-species biofilms. *Enterococcus faecalis* and *Actinomyces naeslundii* were cultured as mono- and dual-species biofilms for 14 days. Crude extracellular polymeric substances (EPSs) from biofilm matrices were extracted using chemical and physical methods. High-performance liquid chromatography, gas chromatography, and mass spectrometry were used to determine the carbohydrate, protein, and fatty acid components. Chemical analysis of the biofilm matrices revealed that they were mainly composed of stachyose, maltose, and mannose carbohydrates. The protein profile in all biofilm samples showed abundant oxidoreductases and chaperone proteins and some virulence- associated proteins mainly located in the membrane surface. High percentages of saturated and monounsaturated fatty acids were identified in all biofilm matrices, with a major prevalence of palmitic, stearic, and oleic acids. Based on the results, it was possible to obtain for the first time a general overview of the biochemical profile of endodontic biofilm matrices.

## Introduction

Endodontic disease such as primary and persistent/secondary infections are clearly biofilm-associated pathologies [1]. Several morphological studies of the root canal in untreated and treated teeth have provided evidence for the presence of self-organized bacterial communities colonizing both the internal and external surface of the root canal system [2, 3]. This is clinically relevant because biofilms are well known to be resistant to the majority of antimicrobial agents and disinfectant protocols used during root canal treatment.

Clinical studies have shown that the biofilm community in root canals is, in fact, a polymicrobial infection. Sundqvist showed that the most common genera in primary infection were *Bacteroides* spp., *Parvimonas* spp., *Lactobacillus* spp., *Actynomyces* spp., and gram-negative anaerobic rods [4]. However, microbiota from persistent/secondary infections differs significantly from that of primary infections. Diversity is very restricted, and the community can move toward a dual or mono-species composition [1]. Enterococcus faecalis an Actinomyces spp. represent two of the species most frequently isolate from persistent root canal infections [2,3]. Both are able to form biofilm, which confers to them the capacity to persist despite adverse environmental root canal conditions [4]. *E*. *faecalis* represents a species that can coexist with other members of the root canal system but is also able to survive as a single organism [5].

Biofilm communities are characterized by the presence of cells firmly attached to and embedded in a self-produced matrix of extracellular polymeric substances (EPSs) [6]. As previously mentioned, bacteria living within biofilm are highly resistant not only to anti-microbial but also to phagocytosis and host immunity [7]. Much of this resistance can be attributed to the matrix EPS, which confers protection through a sophisticated physical barrier against chemical and cellular attack [8]. The structure and components of EPS can be diverse depending on the species of bacteria, environmental conditions, nutrient availability, etc. In general, EPSs are comprised of polysaccharides, extracellular proteins, extracellular DNA (eDNA), lipids, and mainly water [9]. In addition to their protective role, EPSs serve other important functions in the biofilm community; for instance, EPSs mediate biofilm adhesion to surfaces, provide mechanical and structural stability, act as nutrient sources, facilitate quorum sensing and gene transfer, and keep biofilms highly hydrated [10].

Thus, the production of EPSs is essential for biofilm formation, stability, and architecture and influences directly the microbial activity. The characterization of EPSs from the relevant organisms present in endodontic infections is important for gaining a better understanding of the structure-function interactions in biofilms. Anti-biofilm strategies using different agents and therapies (chemical, natural, nanoparticles, photodynamic, etc.) have been extensively studied [11–13]. However, the dynamics of endodontic pathogens and their EPS synthesis are not fully understood. Until now, EPS information from endodontic bacteria has been scarce. Consequently, there is a lack of knowledge about the main components of biofilm matrix produced by the key organisms in root canal infections.

To develop new therapeutic strategies to control initiation, stabilization or dispersal of biofilms, it is crucial to elucidate the main components of biofilm EPSs in detail. Given the importance of EPSs and the limited information available, the main objective of this study is to identify the main biochemical components of biofilm matrix from two gram-positive endodontic pathogens.

## Materials and methods

### Bacteria and culture conditions

Clinically isolated *E*. *faecalis* and *A*. *naeslundii* strains from persistent endodontic infections were used following a previously described protocol [14]. Briefly, after isolation and disinfection of the tooth, cavity access was made with a sterile bur. Following access preparation, microbial sampling was performed using a sterile H-file (Dentsply Maillefer, Tulsa, OK, USA) and sterile paper points (Colténe, Altstätten, Switzerland). The metallic portion of the file, the gutta-percha, and the paper points were transferred into the tube with thioglycolate. Samples were incubated inside an anaerobic chamber (85% N_2_, 10% H_2_, 5% CO_2_) (Coy Laboratory Products, Grass Lake, MI, USA). Identification was performed using specific agar media and API biochemical tests. The confirmation of the identity and antibiotic resistance was performed with the VITEK 2 automated system (BioMeriéux, Cambridge, MA, USA). The samplings of bacteria were from patients who were referred to the Graduate Endodontics Clinic, University of San Luis Potosi, Mexico and approved by the Institutional Ethics Committee of the Faculty of Dentistry at San Luis Potosi University, San Luis Potosi, México (Approval Code CEI-FE-011-014). Informed consent was obtained from the patients after the nature of the procedure, possible discomfort and risks had been fully explained.

### Biofilm formation

Biofilm assays were conducted based on a previously reported method [15]. After confirmation of the strain purity by Gram stain and colony morphology, a single colony of each bacterium was inoculated into 10 mL of brain heart infusion medium (BHI; Becton Dickinson, Hunt Valley, MD, USA) and cultured overnight at 37 °C. The cell density of a monospecies suspension was 10^9^ cells/mL [optical density (OD) = 1] (Ultrospec III; Pharmacia LKB, Piscataway, NJ, USA). A mixed microbial suspension was prepared in a sterile Falcon tube by mixing equal volumes of *E*. *faecalis* and *A*. *naeslundii* suspensions (OD = 1).

For biofilm formation, 1.8 mL of sterile BHI medium and 0.2 mL of inoculum were dispensed into sterile 24-well polystyrene plates. The culture plates were incubated at 37 °C under static anaerobic conditions for 14 days. The growth media were replaced every other day to remove dead cells and provide nutrients. Assays were carried out in triplicate to obtain enough samples for each individual experiment.

### Extracellular polymeric substances extraction

Crude EPS samples were prepared from culture supernatants by the extraction method previously described by Bales et al. (2013), with slight modifications. Briefly, biofilms were repeatedly washed with 0.9% NaCl before harvesting and then fixed with formaldehyde (36.5%) at 4 °C for 1 h. To extract EPS samples were incubated with 0.3 M NaOH at 4 °C for 3 h. Samples were centrifuged (10,000 × g, 4 °C, 20 min), filtered through a 0.2-μm membrane and stored at -20 °C until further analysis [16].

### Saccharide assay

To obtain pure saccharides, proteins and nucleic acids were precipitated with 20% trichloroacetic acid (4 °C, 20 min), and the supernatant was then precipitated twice with two volumes of 96% ethanol (4 °C, 24 h) to recover saccharides. Samples were analyzed by high-performance liquid chromatography (HPLC) using an Agilent Technologies (Santa Clara, CA, USA) LC system equipped with a 1260 Infinity quaternary pump (61311C), a column compartment (G1316A), an automatic liquid sampler module (G7129A), and a refractive index detector (G1362A). Isocratic analysis was performed at 0.8 mL·min^−1^ using water [type I, 0.055 μS·cm^−1^ at 25 °C, 5 μg·L^−1^ total organic carbon, obtained using an A10 Milli-Q Advantage system and an Elix 35 system (Merck KGaA, Darmstadt, Germany)], with 10 μL of sample injected into the system. We performed a complete chromatographic run for glucose under 40 min using a Hi-Plex Ca chromatographic column (P/N PL1170-6810, 7.7 × 300 mm, 8 μm; Agilent Technologies) for analytical separation. The column compartment temperature was kept isothermally at 65 °C.

### Protein assay

The protein profile was determined by loading protein extract samples from TCA precipitate on a 10% polyacrylamide gel for sodium dodecyl sulfate polyacrylamide gel electrophoresis (SDS-PAGE). A prestained protein marker-brand range (PageRuler; Thermo Scientific, Rockford, IL, USA) was used as a molecular weight standard. SDS-PAGE gels were run at 150 V for 1 h. At the end of electrophoresis, the gels were stained overnight in Coomassie blue stain [0.25% (w/v) Coomassie Blue R-250 (Bio-Rad, Hercules, CA, USA), 50% (v/v) methanol and 10% (v/v) acetic acid]. The excess stain was washed out by destaining the gel with a 25% (v/v) methanol solution and 10% (v/v) acetic acid. Finally, the gel was washed with deionized distilled water, photographed, and stored. The most intense bands were excised from and sliced into small pieces using a sterile scalpel. Gel bands were subjected to reduction with dithiothreitol (10 mM) and alkylation with iodoacetamide (50 mM), followed by overnight trypsin digestion (in 25 mM ammonium bicarbonate, 10% acetonitrile) in an automated processor (ProGest; Digilab, Hopkinton, MA, USA).

The resulting tryptic peptides were analyzed by matrix-assisted laser desorption/ionization–tandem time-of-flight (MALDI-TOF/TOF) mass spectrometry (MS) on an AB4800-Plus proteomics analyzer (Applied Biosystems, Foster City, CA, USA). One microliter of a 1:1 mixture of saturated α-cyano-4-hydroxycinnamic acid (in 50% acetonitrile with 0.1% trifluoroacetic acid) and each peptidic sample (1 μL) were spotted onto an Opti-TOF 384 plate, dried, and analyzed in a positive reflector mode. Spectra were acquired using a laser intensity of 3,000 and 1,500 shots/spectrum. CalMix-5 standards (AB Sciex, Redwood City, CA, USA) spotted on the same plate were used for external calibration. From each MS spectrum, up to 10 precursors were submitted to automated collision-induced dissociation MS/MS acquisition at 2 kV in a positive reflector mode (500 shots/spectrum; laser intensity, 3,000).

### Fatty acid methyl ester analysis

Samples for fatty acid methyl ester (FAME) analysis were obtained from crude EPSs. First, lipids were saponified by adding 5.0 mL of 3.75 NaOH mol L-1 of a H_2_O/MeOH (1:1) solution to each sample, followed by mixing, heating in a 100 °C water bath for 5 min, mixing again, and heating in the water bath for an additional 25 min. Then, methylation was carried out in a water bath set at 80 °C for (10 ± 1) min, and rapidly cooled in ice water, using a MeOH/aqueous HCl 6 mol L-1 (3.25:2.75) solution. FAMEs were extracted from this solution by adding 1.5 mL of methyl *tert*-butyl ether:hexane (1:1, v/v) and placing the closed tubes on a rotary mixer for 10 min. The top organic phase was transferred to a test tube and then washed with 3.0 mL of diluted NaOH. The organic phase was then transferred to a gas chromatography (GC) vial for subsequent analysis by GC-MS.

Qualitative analyses of volatile compounds were carried out using a gas chromatographer (Agilent Technologies), equipped with an Agilent Technologies J&W DBWAX microbore column (10 m × 0.1 mm, 0.1-μm film thickness) and an Agilent 5977E mass spectrometer. The carrier gas was helium at a constant flow of 0.3 mL/min. The GC oven temperature was held at 50 °C for 0.34 min, then programmed to 200 °C at a rate of 72.51 °C/min, held at 200 °C for 0.17 min, programmed to 230 °C at a rate of 8.7 °C/min, and held at 230 °C for 7.9 min, for a total run time of 13.93 min. The split ratio was adjusted to 30:1. The injector temperature was set at 250 °C. The mass range was 50–450 m/z. The electron energy was set at 70 eV, 150 °C. Fatty acids were identified using fatty acid methyl ester standards and grouped in classes: saturated, monounsaturated, and polyunsaturated fatty acids. Only hits with a match factor of more than 80% were considered.

### Data analysis

For protein identification, the resulting spectra were searched against the UniProt/SwissProt database using ProteinPilot v.4 and the Paragon algorithm (AB Sciex) at ≥ 95% confidence interval or interpreted manually. A few peptide sequences with lower confidence scores were manually searched using BLAST (http://blast.ncbi.nlm.nih.gov). Predictions of the identified proteins subcellular localization were performed by web-based application SOSUI GramN [17]. VirulentPred was employed to predict the virulence factors among identified bacterial proteins, and the predictions were derived from the Cascade SVM (Support Vector Machine) module [18].

Prevalence of the biofilm EPS components was recorded as the percentage of samples examined. Attempts were made to correlate the components found in the sample concomitantly with its potential producer species.

## Results

### EPS saccharide profile

The monomeric composition of purified exopolysaccharides was determined by HPLC. The main identified carbohydrates were stachyose, mannose (Man), and maltose (Mal). The oligosaccharide stachyose showed elevated concentrations in all EPS samples. Biofilm EPS groups gave rise to variations in sugar composition. Table 1 shows the overall expression of the biofilm EPS saccharide profile.

**Table 1:** HPLC Data of the saccharides analyzed.

Saccharide	Retention time (min)	Biofilm *E*. *faecalis*	Biofilm *A*. *naeslundii*	Biofilm Dual species
**Stachyose**	6.385	5.75^a^	8.55	4.55
**Maltose**	7.068	2.98	1.36	0.18
**Lactose**	7.247	1.14	ND	ND
**Mannose**	7.625	3.26	1.54	1.19
**Glucose**	8.069	0.03	0.05	ND
**Xylose**	9.124	1.28	ND	ND
**Galactose**	9.233	0.09	ND	ND
**Fucose**	10.795	0.13	3.57	ND
**Ribose**	18.481	ND^b^	0.13	0.09

^a^Values expressed as mmol/mL concentration.

^b^Not detected.

### EPS protein profile

By visual inspection of the patterns of the EPS proteins from all the examined species, 45 bands were observed. Typically, three major bands were seen at 10–20 and 30–50 kDa, respectively. Table 2 shows the MALDI-TOF-TOF MS identified proteins in all samples. We characterized the identified proteins according to their function and cellular location. Based on the prediction system SOSUI [17], the majority of the proteins originated from the cytoplasm. Molecular functions of the identified proteins were extracted from the UniProt database, which mostly revealed participation in metabolic processes, with pathways related to carbohydrate, amino acid, and nucleotide/nucleic acid metabolism. Proteins associated with lipid biosynthesis and membrane location were classified as virulent, using VirulentPred [18], a prediction method based on the cascade support vector machine (SVM) module. Another membrane protein associated with biofilm formation was identified in *E*. *faecalis* matrix, ornithine carbamoyltransferase.

**Table 2:** Main proteins identified in the mono- and dual-species biofilm.

Accession	Protein Description	Gene Name	Major Function	Localization
***E*. *faecalis***				
**P13794**	Outer membrane porin F	*oprF*	Porin	Membrane
**R2RVY7**	Glyceraldehyde-3-phosphate dehydrogenase	*UAK_01214*	Oxidoreductase	Cytoplasmic
**J6CBD5**	Pyruvate kinase	*HMPREF1335_02348*	Kinase	Cytoplasmic
**Q8E3E7**	Elongation factor G	*fusA*	Elongation factor	Cytoplasmic
**Q839G8**	Elongation factor Tu	*Tuf*	Elongation factor	Cytoplasmic
**P0DM31**	Enolase	*Eno*	Lyase	Membrane
**Q93K67**	Arginine deiminase,	*arcA*	Hydrolase	Cytoplasmic
**Q1GB26**	Phosphoglycerate kinase	*Pgk*	Kinase, transferase	Cytoplasmic
**Q839Q5**	Ornithine carbamoyltransferase (OTCase), catabolic	*arcB*	Transferase	Cytoplasmic
**Q9MTM3**	DNA-directed RNA polymerase subunit beta	*P027_00306*	Oxidoreductase	Cytoplasmic
**A8MHG9**	30S ribosomal protein S2	*rpsB*	Ribonucleoprotein	Cytoplasmic
**J5JNN7**	Phosphate acetyltransferase	*HMPREF1342_00604*	Acyltransferase	Cytoplasmic
**J6CLQ9**	PTS system mannose-fructose-sorbose IID component	*HMPREF1335_01036*	Transmembrane	Membrane
**L2L4B1**	D-alanyl-lipoteichoic acid biosynthesis protein DltB	*OIE_05783*	Virulence	Membrane
**P21310**	Histidine ammonia-lyase	*hutH*	Lyase	Cytoplasmic
**J6RL23**	Peroxiredoxin	*HMPREF1342_00219*	Oxidoreductase	Cytoplasmic
**Q838I4**	Superoxide dismutase [Fe]	*sodA*	Oxidoreductase	Cytoplasmic
**WP_031798994**	Major outer membrane lipoprotein	*oprI PA2853*	Virulence	Membrane
**A0A219BJA5**	Alpha-L-fucosidase	*B645_08885*	Alpha-L-fucosidase activity	Cytoplasmic
***A*. *naeslundii***				
**P13794**	Outer membrane porin F	*oprF*	Porin	Membrane
**G9PDB9**	Uncharacterized protein	*der*	Kinase	Cytoplasmic
**A0A242CXV1**	Uncharacterized protein	*A5875_004254*	Unknown	Unknown
**S2WED7**	Putative glutamate—cysteine ligase 2	*HMPREF9238_00733*	Ligase	Cytoplasmic
**B7VMI4**	Holliday junction ATP-dependent DNA helicase RuvB	*ruvB*	Helicase, hydrolase	Cytoplasmic
**A0A2I1KRB6**	Peptidyl-prolyl cis-trans isomerase	*CYJ26_08890*	Isomerase	Cytoplasmic
**A0A242C5U1**	Protein translocase subunit SecA	*secA*	Translocation	Membrane
**A0A242CXV1**	Uncharacterized protein	*A5875_004254*	Unknown	Unknown
**A0A095WN18**	Alpha-ketoglutarate decarboxylase	*kgd*	Lyase	Cytoplasmic
**A0A1Q5Q2K4**	Shikimate dehydrogenase (NADP(+))	*aroE*	Oxidoreductase	Cytoplasmic
**P11221**	Major outer lipoprotein	*oprI PA2853*	Virulence	Membrane
**SHE26639**	Peptidyl-prolyl cis-trans isomerase	*ACGLYG10_2892*	Isomerase	Cytoplasmic
**A0A1Q5PWS3**	Lysine—tRNA ligase	*lysS*	aminoacyl-tRNA synthetase	Cytoplasmic
**Dual species**				
**A6V748**	Outer membrane protein OprF	*oprF*	Porin	Membrane
**G9PGG6**	DNA gyrase subunit A	*gyrA*	Isomerase	Cytoplasmic
**P13794**	Outer membrane porin F	*oprF*	Porin	Membrane
**A0A1M6FM65**	Polyribonucleotide nucleotidyltransferase	*pnp*	Transferase	Cytoplasmic
**A0A242CXV1**	Uncharacterized protein	*A5875_004254*	Unknown	Unknown

### EPS fatty acids profile

**Fig 1:**
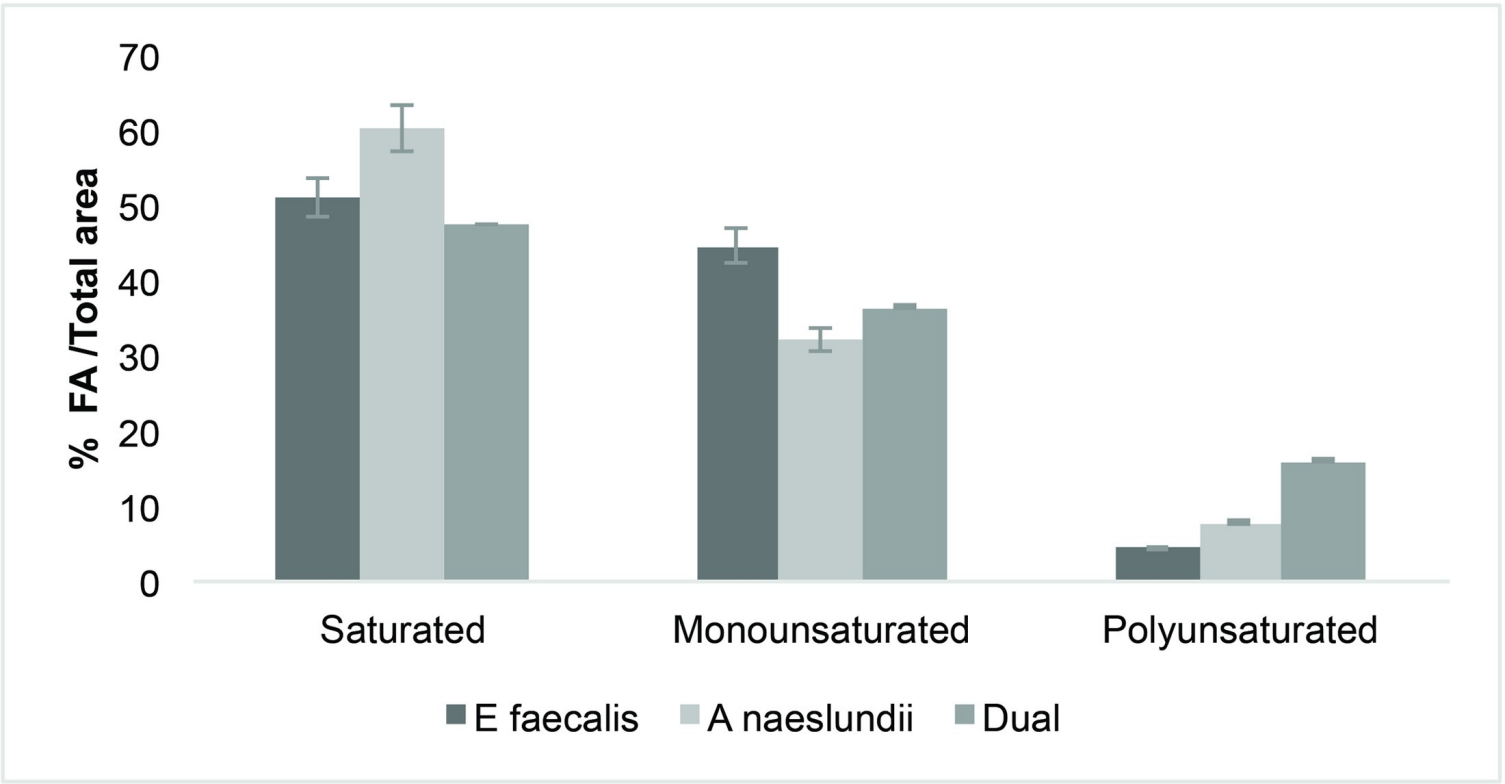
Fatty acid composition for biofilm matrices of *E*. *faecalis*, *A*. *naeslundii*, and dual species. A high percentage of saturated and monounsaturatred fatty acids was observed in the three biofilm samples.

After fatty acid extraction (MIDI protocol), 10 fatty acids (FAs) in the form of their methyl esters were identified in the EPS samples of the biofilm species (Table 3). Palmitic (16:0), stearic (18:0), and oleic (18:1n9c) acids represented the major fractions from the three biofilm EPS samples. Acetic (2:0), butyric (4:0), succinic (4:0 diacid), lauric (12:0), myristic (14:0), palmitoleic (16:1n9c), and pentadecanoic (15:0) acids accounted for the minority of FAs in the EPS matrix. Based on the classification, biofilm matrices exhibited a high percentage of saturated and monounsaturated fatty acids (Fig 1).

**Table 3:** Main fatty acids from the mono- and dual-species biofilms.

Fatty Acid	Shorthand	*E*. *faecalis*	*A*. *naeslundii*	Dual species
**Acetic**	2:00	9,43 ± 0,125^a^	ND^b^	ND
**Butyric**	4:00	7,22 ± 0,036	ND	ND
**Succinic**	4:0-diacid	1,56 ± 0,081	8,51 ± 0,081	ND
**Lauric**	12:00	5,66 ± 0,134	ND	ND
**Myristic**	14:00	3,17 ± 0,165	6,28 ± 0,165	3,60 ± 0,155
**Pentadecanoic**	15:00	1,72 ± 0,011	3,83 ± 0,332	0,162 ± 0,0125
**Palmitic**	16:00	22,62 ± 0,44	2,03 ± 0,341	24,18 ± 0,947
**Stearic**	18:00	14,6 ± 0,351	17,19 ± 0,36	ND
**Palmitoleic**	16:1n9C	1,14 ± 0,03	ND	ND
**Oleic**	18:1n9c	17 ± 0,251	18,81 ± 0,406	14,26 ± 0,288

^a^Values represent the means ± SD

^b^Not detected

## Discussion

Bacterial biofilms are commonly associated with the establishment of apical periodontitis. These bacteria within biofilms are encased in a highly organized matrix, which plays a critical role in the biofilm resistance [8]. The EPS composition of this matrix varies mainly according to the species, environmental conditions, and nutrient availability [19]. Hence, we present an approach to ascertaining the principal components (polysaccharides, proteins, and fatty acids) of biofilm matrices associated with two endodontic pathogens. The data available about the EPSs produced by these bacteria is scarce. Thus far, the closest related information has been provided by a few clinical studies that described the protein and fatty acid content found in root canal samples [20–22].

Polysaccharides in the extracellular matrix have been found to be an important structural component in biofilms [23]. The presence of diverse monosaccharides and linkages in bacterial extracellular polysaccharides alter carbohydrate chemical analyses in a very interesting but challenging way. Most of the saccharides found in the present study, mannose (Man), glucose (Glc), galactose (Gal), and ribose (Rib), are commonly described as part of bacterial exopolysaccharides. The identification of Glc, Gal, Man, and Rib in the *E*. *faecalis* matrix have previously been reported. Hancock and Gilmore identified a polysaccharide containing Glc, Gal, rhamnose (Rha), glucosamine (GlcN) and phosphate (Pho) [24]. Pazur reported a tetraheteroglycan from the cell wall of a strain of *E*. *faecalis* with a similar composition [25]. Nonetheless, in this study, we found that stachyose, an oligosaccharide with an intestinal regulatory function, was a major constituent of the biofilm matrix. Stachyose is a tetrasaccharide composed of two D-galactose units, one D- glucose unit, and one D- fructose unit sequentially linked. It belongs to the commonly named raffinose family oligosaccharides (RFOs), and is known as an important carbohydrate transport [26]. So far, what has been described in the literature in relation to ROF components raffinose-induced biofilm formation by *S*. *mutans* in a mixed culture with sucrose [27]. Further, it has been reported that contribute to the extracellular polysaccharides production in some oral bacteria [28]. In a previous study, stachyose was designated as a carbon source utilized by the microbial community in a group of patients with periodontitis [29]. Though the involvement of stachyose in *E*. *faecalis* or *A*. *naeslundii* biofilm matrix is unknown, our findings suggest it is an important component of the EPSs. Further research is needed to gain a better understanding of the mechanism underlying the role of ROF in biofilm formation.

Previous studies of *Actinomyces* spp. polysaccharides indicated that N-acetylglucosamine was the major component, while Gal, Man, Glc, uronic acid, and smaller amounts of glycerol (Gro), Rha, and xylose (Xyl) were also present [30]. Yamane et al. performed a chemical analysis of the EPS matrix of *A*. *oris* isolated from an apical abscess lesion and revealed that it was mainly composed of Man, Glc and Rib. These results are consistent with ours, since these three were the main saccharides found in the *A*. *naeslundii* EPS. The authors also concluded that the expression of this phenotype might play an important role as a virulence factor in the establishment of apical periodontitis [31].

The protein profile obtained from the two-species biofilm is mainly related to metabolic and housekeeping processes, including metabolism energy and protein synthesis. As Provenzano J et al. [20] stated, this is indicative of a living active microbial community. It has been reported that the presence of an enzymatically active form of glyceraldehyde-3-phosphate dehydrogenase (GADPH), either secreted or cell surface located, can be associated with the possibility of multiple extracellular functions [32]. This is especially relevant for moonlighting proteins [33] and for the secreted glycolytic enzyme enolase. Both cell surface-associated, multitasking proteins, GADPH and enolase identified in our study can be virulent and play an important role in interactions with the host, including adaptive responses to environmental changes, adherence, internalization, synthesis of toxins, and avoidance of the host immune system [33,34]. Enolase has been described as a surface associated protein in most streptococci species and shows properties like plasminogen-binding. [35]. Furthermore, the presence of oxidoreductases and chaperone proteins in oral biofilms has been associated with inflammation [36]. Another relevant identified protein is ornithine carbamoyltransferase (OTCase), which is associated with biofilm formation [37].

We performed a prediction of virulence factors with the VirulentPred [18] tool from the cascaded SVM module. EPS proteins with a higher virulence potential were mainly uncharacterized proteins, histidine ammonia-lyase, and lipoproteins (Lpps) from mono- and dual-species biofilms. Similar to the function of lipopolysaccharides in gram-negative bacteria, Lpps is a gram-positive bacterium that performs an important function in immune regulation [38,39]. Although it is well known that they play a crucial role in pathogenicity, additional information is needed to understand the multiple effects of Lpps in gram-positive bacteria.

In this study, we also investigated the FA composition of biofilm EPSs. The occurrence of FAs in infected root canals in a previous study suggests that these molecules may have a role as a virulence factors in the pathogenesis of apical periodontitis [19]. Our results showed that SFAs (palmitic, stearic, and oleic) were the most common in mono and dual-species EPSs, followed by smaller quantities of short-chain fatty acids (SCFAs) (acetic, butyric, and succinic) that were identified only in mono species biofilms. Both, SFAs and SCFAs have been associated with cytotoxicity, inflammation, and suppression of the immunological response [40,41]. For instance, butyric acid inhibits T-cell proliferation, and apoptosis in monocytes and lymphocytes. Succinic acid inhibits neutrophils and phagocytic activity [42]. As previously mentioned, FA profiles of biofilm EPSs showed a higher SFA content. Thus, biofilm exhibits a physiological state, which tends to decrease membrane fluidity, thereby limiting interactions between bacteria and their environment. It has been proposed that increased membrane saturation in biofilm is an adaptive stress response that provides advantages to the bacteria within biofilm, over their planktonic counterparts. Additionally, this change in fluidity may contribute to their high tolerance for adverse environmental conditions, including antimicrobial therapy.

Although bacteria biofilm model used in the current investigation could represent a limited example given the polymicrobial nature of root canal infections. As was mentioned before, Gram positive bacteria such as *E*. *faecalis* and *A*. *naeslundii* are clearly involved in the development of root canal persistent/secondary infection [43]. In this sense, the biofilm model used in this study works as a valid source of information in a field so far little studied.

## Conclusions

Here, we present the first description of the main biomolecules potentially released by endodontic pathogens that are components of the biofilm matrix present in persistent/secondary endodontic infections. The information provided highlights the relevance of further identification and investigation of these biomolecules as possible virulence factors and targets of anti-biofilm therapeutic agents during root canal treatment.
